# From Gaze Allocation to Scan Sequences: A Segment-Sensitive Eye-Tracking Study of Pilot Scan Discipline During Simulated Instrument Landing System Approaches

**DOI:** 10.3390/jemr19050109

**Published:** 2026-09-23

**Authors:** Bo Fu, Jonathan Peña, Oscar Arenas, Russell Harral

**Affiliations:** Department of Computer Engineering and Computer Science, California State University, 1250 Bellflower Blvd., Long Beach, CA 90840, USA; jonathan.pena01@student.csulb.edu (J.P.); oscar.arenas01@student.csulb.edu (O.A.); russell.harral01@student.csulb.edu (R.H.)

**Keywords:** human factors in aviation, empirical studies, human–computer interaction

## Abstract

Instrument Landing System (ILS) approaches impose distinct visual demands across two operational segments, including (i) an intermediate approach segment prior to the final approach fix (FAF), during which pilots intercept and track the localizer while complying with published altitude restrictions and the glideslope-intercept altitude, and (ii) the final approach (FA) segment from the FAF to decision altitude/decision height, during which pilots precisely maintain both localizer and glideslope guidance. Using eye tracking, this study examines segment-specific scan behavior during simulated ILS approaches in low visibility conditions performed by 36 licensed pilots in a Cessna 172 equipped with G1000 avionics. The study results show that successful approaches were characterized by suppressed external viewing, more compact scans, and more organized return-loop patterns, suggesting that effective ILS scan discipline depends not only on looking at task-relevant instruments, but also on organizing those scans into phase-appropriate and functionally integrated sequences. By linking gaze allocation, scan dispersion, and scan sequence patterns to approach outcomes, this study demonstrates how eye tracking can provide segment-sensitive markers of instrument-scan quality. We further propose candidate scan-assessment metrics that may contribute to more precise and objective simulator debriefing, as well as future flightdeck-interface concepts that may support disciplined cross-checking without increasing pilot workload.

## 1. Introduction

Eye tracking has been increasingly adopted as a useful method for examining visual attention and gaining insight into pilots’ scanning behavior across a range of aviation contexts [1,2]. Gaze analysis is particularly relevant to instrument flight, during which pilots must continuously sample and integrate information from multiple flightdeck displays while operating with limited external visual references. Within this broader setting, Instrument Landing System (ILS) approaches offer a useful case for investigating how gaze behavior evolves as pilots progress through operationally distinct flight segments with changing visual demands. Using eye-tracking data from 36 licensed pilots, we compare segment-specific gaze behavior in successful and unsuccessful ILS approaches and quantify differences in fixation behavior, saccadic activity, gaze allocation across flightdeck areas of interest, and transitions between key instruments. This segment-based analysis identifies eye-movement patterns associated with effective scan discipline, shows how performance-relevant visual strategies vary across the approach, and provides a phase-sensitive account of pilot instrument monitoring with direct relevance for assessment, simulator debriefing, training interface and flightdeck design. In this paper, scan discipline refers to the phase-appropriate organization of visual sampling across task-relevant flightdeck information sources, such as where gaze is allocated, how spatially dispersed it is, and how instrument checks are sequenced and reintegrated over time. As such, scan discipline differs from gaze allocation alone, since allocation describes where or for how long a pilot looks, whereas scan discipline additionally concerns whether those observations form an organized and operationally appropriate cross-check.

## 2. Related Work

Eye tracking is widely used in aviation research to examine how pilots allocate visual attention during routine and high-workload operations [1,2]. In a review of 50 aviation eye-tracking studies, Ziv [1] identified consistent differences between expert and novice pilots, including more efficient scan organization, better use of peripheral information, and greater attention to task-relevant instruments. Peißl et al. [2], in a review of 79 studies, grouped aviation eye-tracking applications into three broad areas: understanding human performance, informing display design, and assessing physiological factors that influence pilot behavior. Across this literature, commonly reported measures include fixation duration, saccade length and frequency, pupil diameter, blink rate, and microsaccades. These measures have been used to generate heat maps, scanpaths, and gaze-transition models, supporting the analysis of pilot performance, situational awareness, and flight deck usability.

A consistent finding in prior work is that experienced pilots tend to show more organized and task-relevant gaze behavior than less experienced pilots [3,4,5,6]. Compared with novices, they typically exhibit more focused fixations, more efficient scanpaths, and less diffuse visual sampling of flightdeck information. By contrast, ineffective gaze allocation has been associated with poorer performance [7] and with crew errors during demanding phases such as final approach and go-around [8]. More recent work further suggests that pilot expertise shapes visual behavior in low-visibility operations, with experienced pilots showing faster attentional shifts and being more likely to initiate a go-around when conditions warrant it [9]. Ocular measures such as pupil diameter, blink rate, and microsaccades have been studied as indicators of cognitive workload [10,11,12,13] and are increasingly being incorporated into adaptive flightdeck and training systems for real-time monitoring of pilot state [14].

Recent studies have also extended gaze analysis toward prediction and classification. Gaze features such as saccade magnitude and direction, fixation duration, pupil dilation, and blink rate have been used to detect operationally relevant events and support performance prediction [15,16]. Other work has shown that gaze behavior varies systematically across flight phases and can therefore be used to classify pilot task context [17]. Related studies of expertise classification during approach and landing similarly report that expert pilots display more anticipatory and structured gaze behavior, whereas novices exhibit broader and less stable scan patterns [18]. In training contexts, gaze-based feedback has been used to support instruction and debriefing by making attentional strategies observable to instructors and trainees [14,19]. Multimodal systems that combine ocular measures with physiological signals have further expanded this line of work by enabling workload-sensitive training interventions [20], while interface studies have used gaze metrics to evaluate cockpit usability and cognitive demand [21].

Contributing to this existing body of knowledge, this paper extends prior research [22] to examine gaze behavior during simulated ILS approaches with an emphasis on segment-specific scan organization and compares successful and unsuccessful performance within this structure. This study design allows us to ask not only whether gaze behavior differs as a function of outcome, but also how scan discipline changes as the task demands shift from localizer interception and altitude management to coupled lateral and vertical guidance on the final approach. Using fixation-based, saccadic, area-of-interest, and transition-based measures, this study identifies gaze features associated with successful ILS approaches and proposes segment-sensitive metrics that are directly relevant to pilot assessment, simulator debriefing, and training interface and flightdeck design.

## 3. Eye-Tracking Study

To examine whether successful and unsuccessful ILS approaches are associated with different patterns of scan discipline, we conducted a controlled eye-tracking study in a simulated environment. It is relevant to note that instrument scan behavior is grounded in established principles of attitude-instrument flying. The FAA identifies instrument cross-check and instrument interpretation as fundamental skills that jointly support positive aircraft control [23]. Instrument cross-check, also referred to as scanning or instrument coverage, involves the continuous and logical observation of instruments for attitude and performance information. Importantly, FAA guidance does not prescribe a single fixed cross-check sequence, but rather, emphasis should be placed on the instruments providing the most pertinent information for the particular maneuver or phase of flight [23]. Accordingly, scan discipline is not equivalent to maximizing attention to any single instrument. In the context of this paper, we operationalize scan discipline as the phase-appropriate organization of visual sampling across task-relevant flightdeck information sources, encompassing where gaze is allocated, how spatially dispersed it is, and how instrument checks are sequenced and reintegrated over time. In other words, the study presented in this paper is guided by FAA-informed construct-level expectations, e.g., successful performance is expected to be associated with more instrument-centered scanning, better integration of task-relevant flight information, and adaptation of scan organization as task demands changes during approach segments, but the specific metric-level and sequence-level characteristics through which these broader expectations were expressed are examined exploratorily. In this way, the study links FAA-informed principles of instrument cross-check with empirical eye-tracking measures while distinguishing operationally grounded expectations from exploratory identification of specific performance-related gaze patterns.

### 3.1. Study Design

Using the X-Plane 12 Flight Simulator, each pilot performed an ILS approach to the Seattle–Tacoma International Airport (KSEA) in a Cessna 172 equipped with G1000 avionics. At the scenario’s onset, the aircraft was positioned 5 nautical miles south of a designated waypoint at a prescribed heading and altitude. Participants were instructed to intercept the localizer, fly the published procedure, descend on the glideslope, and land the aircraft. Each pilot was given a single attempt and was not permitted to execute a go-around or missed approach. Throughout the task, pilots were instructed to maintain 90 knots and to continuously monitor the flight instruments, making the maneuver dependent on sustained and precise instrument scanning.

The primary instruments required for the task were the Attitude Indicator (AI), Altimeter/Vertical Speed Indicator (Alt_VSI), Airspeed Indicator (ASI), Slip–Skid Indicator (SSI), Turn Indicator/Horizontal Situation Indicator (TI_HSI), and Tachometer (RPM), shown in Figure 1. These displays provide the information necessary for pitch and bank control, altitude and descent-rate monitoring, airspeed control, lateral coordination, navigational alignment, and power management. Successful execution of the approach, therefore, required pilots to integrate information across these instruments to control speed, track the localizer, remain on glideslope, maintain coordinated flight, and manage power. The Flight Director was disabled so that performance depended on manual instrument interpretation rather than command guidance, thereby increasing the relevance of measured scan behavior to the visual–cognitive demands of the task.

The study environment was configured to represent a moderately demanding Instrument Flight Rules approach. Weather conditions included wind from 300° at 18 knots, producing a crosswind component that required active control to maintain localizer alignment. A cloud base of 400 ft above ground level with limited external visual references was set, and visibility was set to 1 statute mile, such that the runway environment would not become visible until late in the descent. The altimeter setting was 29.92 inHg and the outside air temperature was 7 °C. Turbulence was disabled to reduce stochastic aerodynamic disturbances and improve experimental control, allowing performance differences to be interpreted primarily in relation to pilot control and scan behavior rather than environmental variability. Collectively, these settings created a controlled but operationally realistic instrument environment in which pilots needed to maintain lateral and vertical guidance, manage airspeed, and cope with crosswind effects while relying predominantly on the flightdeck instruments.

### 3.2. Defining Success

Consistent with the FAA Instrument Rating Airplane Airman Certification Standards for precision approaches, performance in the present study was evaluated along three dimensions: lateral tracking of the localizer, vertical tracking of the glideslope, and airspeed control. Specifically, the FAA standards specify that pilots should maintain a stabilized final approach from the FAF to DA/DH, with no more than three-quarter-scale deflection of the lateral or vertical guidance and with airspeed maintained within ±10 knots of the target speed [24].

An overall approach score, *p*, was defined as the equally weighted mean of the three components across the full approach. Let *n* denote the number of sampled flight data points, recorded every one-third of a second. At each time point *i*, lateral, vertical, and speed performance values were computed and summed; the overall score was then obtained by averaging these values across all samples, treating lateral guidance, vertical guidance, and energy management as co-equal contributors to approach performance:

(1) $$p=\frac{1}{3n}\sum_{i=1}^{n}\left({lateral}_{i}+{vertical}_{i}+{speed}_{i}\right)$$

For each X-Plane data sample, a maximum of one point was assigned to each scoring component outlined in Equation (1) and out-of-tolerance deviations were penalized. To support outcome-based comparisons, continuous scores were also converted into a binary performance classification. Based on the FAA standard tolerances [24,25,26], as well as consultations with certified flight instructors and FAA-designated pilot examiners, approaches meeting the tolerances should yield *p* ≥ 0.70 (i.e., classified as successful), whereas approaches outside the tolerances would yield *p* < 0.70 (i.e., classified as unsuccessful). This classification was applied to translate continuous measures into a pass/fail outcome in the study to mirror practical instrument evaluations in the real world, in which performance is observed and judged by certified flight instructors against fixed proficiency criteria. As such, this threshold is not intended as a universally applicable proficiency cutoff.

We further divided the overall approach into two segments for analysis, including (i) a pre-FAF segment, during which the pilot intercepted and tracked the localizer while complying with published altitude restrictions or the glideslope-intercept altitude, and (ii) the FA segment, beginning at the FAF, during which the pilot tracked both the localizer and glideslope to DA/DH.

In the pre-FAF segment, localizer performance was scored when the heading and bank angle remained within predefined bounds (heading was within 25° of 344° and the absolute bank angle was less than 15°), with points decreasing as localizer deflection increased, as defined in Equation (2). The vertical performance was scored according to compliance with the applicable minimum altitude, with full credit above the threshold and partial credit for deviations of fewer than 100 feet below it, as defined in Equation (3). Airspeed performance was scored relative to a target speed of 90 knots, with points decreasing linearly within a 10-knot tolerance and no credit assigned outside that range, as defined in Equation (4). The pre-FAF segment score was then obtained by summing the points earned across all samples and dividing by the total number of samples.

(2) $$1-\frac{\left|localizer\;delfection\right|}{1.875}$$

(3) $$1-\frac{minimum\;altitude-altitude}{100}$$

(4) $$1-\frac{\left|90-speed\right|}{10}$$

The lateral (Equation (2)) and airspeed components (Equation (4)) were defined throughout the approach using localizer deviation and deviation from the target airspeed, respectively, whereas the vertical component was segment-dependent. In the pre-FAF segment, it reflected compliance with altitude restrictions (Equation (3)), and in the FA segment, it reflected glideslope tracking (Equation (5)). Specifically, glideslope performance was scored when vertical speed remained greater than −1000 feet per minute, with the score decreasing linearly as glideslope deflection increased.

(5) $$1-\frac{\left|glideslope\;delfection\right|}{1.875}$$

### 3.3. Participants

A total of 50 real-world pilots took part in the study. Five preliminary dry-run sessions were performed with three certified flight instructors and two licensed fixed-wing pilots prior to formal data collection. These dry runs were necessary to refine the simulation parameters, task procedure, and experimental protocol to improve operational realism and experimental control. Data from the dry runs were not included in the final analysis, since these sessions were procedural and technical in purpose rather than a formal statistical pilot study and therefore were not used for inferential testing, effect-size estimation, or a priori sample-size determination. We further excluded incomplete (e.g., missing, corrupted, or truncated) and invalid recordings (n = 9) due to loss of gaze or simulator signals. The final analysis presented in this paper was sampled from 36 pilots with an average proportion of valid gaze samples of 95.8% (SD = 0.03).

Participants held a range of FAA-issued certificates, including student pilot, private pilot, commercial pilot, flight instructor, and airline transport pilot certificates. Reported qualifications also included endorsements and ratings such as instrument, complex, high-performance, and multi-engine. Across the retained sample, participants reported a mean of 1330 total flight hours (SD = 3246), 40 h (SD = 120) of instrument approach experience in actual instrument meteorological conditions, 83 h (SD = 135) in simulated instrument conditions, and 15 ILS approaches (SD = 22) performed in the 6 months preceding the study. In the context of this study, expertise refers to background characteristics such as pilot certification, total flight time, and instrument-flight experience, whereas performance refers specifically to the execution of the experimental ILS approach. Participants were classified according to approach performance rather than expertise, recognizing that substantial flight experience does not necessarily guarantee successful performance in a given approach.

### 3.4. Study Configuration

The study was conducted in a controlled indoor office environment with stable lighting. A simulated ILS approach scenario was presented in X-Plane 12 Flight Simulator, and the physical configuration is shown in Figure 2a. The simulation ran on a workstation equipped with an Intel Core i9 processor, an NVIDIA GeForce RTX 3080 (10 GB), and a 24-inch Full HD display (1920 × 1080 pixels, 60 Hz). Flight control inputs were provided through Logitech G hardware, consisting of rudder pedals, a throttle quadrant with dual levers, a flight yoke, and a trim wheel. Aircraft audio cues and simulated air traffic control communications were presented through wireless headphones.

Eye movements were recorded with a Gazepoint GP3 HD eye tracker using a nine-point calibration procedure. The device sampled at 150 Hz, with a stated accuracy of 0.5–1.0° visual angle and a tracking range of 35 cm × 22 cm with ±15 cm depth tolerance. The setup was refined in consultation with certified flight instructors and was validated against the flightdeck geometry of an actual Cessna 172 equipped with G1000 avionics (Figure 2b), ensuring that the eye tracker’s field of view was compatible with the corresponding real-world angle of incidence. Participants sat on non-wheeled and non-swivel office chairs throughout the study session and were seated at an approximately 65 cm viewing distance from the computer monitor. The seating position, monitor location, and flightdeck display perspective were held constant throughout the experiment.

### 3.5. Data Collection

Before each session, participants provided informed consent and completed a brief eligibility questionnaire. The questionnaire collected factual background information, including licensing status and flight experience in both actual and simulated instrument conditions. Participants then received a standardized task briefing covering the flightdeck instruments, controls, recommended operating speeds, the approach plate, and the relevant weather information for the KSEA scenario. They were instructed to fly the ILS approach as they normally would after receiving the simulated air traffic control clearance. Each participant then completed eye-tracker calibration and remained in a fixed seating position throughout the experiment.

In addition to simulator-derived flight performance variables, we computed a comprehensive set of gaze measures to characterize visual behavior during the approach (Table 1) using BEACH-Gaze [27], including fixation-, saccade-, blink-, pupil-, and scanpath-based metrics. Where applicable, descriptive summaries such as the sum, mean, median, standard deviation, minimum, and maximum were also generated to characterize central tendency and variability. Peak saccadic velocity was computed using the method proposed in [28], and saccade amplitude was calculated as Euclidean distance. Additional descriptive measures included blink rate, pupil size and pupil dilation for each eye and across both eyes, fixation-to-saccade ratio, and total scanpath duration. Pupil dilation was defined relative to a baseline pupil size obtained during the calibration procedure before task onset. Convex hull was defined as the smallest area that encompasses all fixations, providing a measure of the spatial dispersion of the fixation set.

We also computed AOI-specific measures (Table 2) as shown in Figure 1, including stationary entropy, reflecting the distribution of gaze across AOIs, and transition entropy, reflecting the degree of randomness in gaze shifts between AOIs [29,30]. Further AOI measures included the proportion of fixations and fixation duration allocated to each AOI, as well as pairwise transition counts and transition proportions. Transitions were treated as directional, such that AOI_A→AOI_B was considered distinct from AOI_B→AOI_A. Self-transitions were defined as successive returns to the same AOI without an intervening visit to another AOI.

To characterize scan structure at the sequence level, we extracted ordered AOI sequences and recurring subsequence patterns using the procedures described in [31,32]. Patterns were represented in both expanded and collapsed forms. Expanded sequences retained repeated consecutive fixations within the same AOI, whereas collapsed sequences removed such repetitions. For example, the sequence ACCDEABAAAABC was reduced to ACDEABABC in collapsed form. For each pilot, we then computed pattern frequency, sequence support, mean pattern frequency, and proportional pattern frequency relative to the full dataset.

## 4. Results

Between-group differences were evaluated using independent-sample t tests, and associations between gaze measures and continuous performance scores were examined using Pearson correlations. Statistical significance was evaluated at *α* = 0.05. Effect sizes are reported with 95% confidence intervals, using Hedges’ *g* for between-group comparisons and Pearson’s *r* for gaze–performance associations. Note that Pearson’s correlation should be interpreted as association rather than causation. We applied the Benjamini–Hochberg (B-H) false discovery rate procedure to multiple comparisons (*q* < 0.05). Correlation analyses were included because the approach score was defined as a continuous variable, allowing gaze–performance relationships to be examined across the full range of outcomes. In parallel, the binary classification of successful versus unsuccessful approaches was retained to reflect the pass/fail logic of practical flight evaluation and to support training-relevant group comparisons.

Of the 36 pilots, 22 were classified as successful and 14 as unsuccessful. The successful group included 18 instrument-rated pilots with an average of 1896 total flight hours (SD = 4060), 57 h (SD = 151) in actual instrument conditions, 109 h (SD = 166) in simulated instrument conditions, and 17 ILS approaches (SD = 26) flown in the 6 months preceding the study. The unsuccessful group included eight instrument-rated pilots, with an average of 440 total flight hours (SD = 588), 12 h (SD = 26) in actual instrument conditions, 43 h (SD = 37) in simulated instrument conditions, and 13 ILS approaches (SD = 14) in the preceding 6 months.

### 4.1. Approach Performance–Gaze Tendencies During the Pre-FAF Segment

Figure 3 shows statistically significant strong (1.0 ≥ |r| ≥ 0.6) and moderate (0.6 > |r| ≥ 0.4) correlations observed between gaze measures and the pre-FAF segment scores that remained statistically significant after B–H false discovery rate correction, ranked by strength. The associations were dominated by negative relationships with OTW gaze measures, with the strongest associations observed for OTW sum of peak saccade velocity (r = −0.781), OTW sum of relative degrees (r = −0.778), OTW sum of mean saccade velocity (r = −0.768), OTW sum of saccade amplitudes (r = −0.767), and OTW sum of saccade lengths (r = −0.766). Higher pre-FAF performance was also associated with lower proportions of fixations (r = −0.743) and duration (r = −0.740) allocated to the OTW, shorter OTW scanpath duration (r = −0.740), lower dwell time in OTW (r = −0.738), fewer fixations (r = −0.727) and saccades (r = −0.725) in OTW, lower summed saccade duration (r = −0.686) and lower summed absolute gaze-direction change in the OTW region (r = −0.635). These negative associations suggest that higher-performing pilots allocated less visual attention to the OTW during the pre-FAF segment. This pattern is consistent with the operational demands of the pre-FAF segment, during which pilots must intercept and track the localizer, comply with altitude constraints, and maintain the target airspeed primarily through instrument reference. Given the limited visibility and the absence of useful runway visual references until later in the descent, greater OTW sampling during this segment may reflect less task-relevant visual allocation.

Positive correlations further characterize the scan behavior associated with higher pre-FAF performance, such as greater maximum relative gaze-direction change (r = 0.623) and greater ASI-related directional change evidenced through mean and median relative degrees (r = 0.550, r = 0.539 respectively). These associations suggest that higher-performing pilots may have made more pronounced gaze transitions when integrating task-relevant instrument information, particularly information related to airspeed control.

Overall, these results motivate a segment-specific interpretation of scan discipline. The concentration of significant negative associations within the OTW AOI suggests that excessive external visual sampling may characterize less effective scan discipline during this phase of the approach. Conversely, the positive associations with gaze directions may reflect more deliberate transitions among task-relevant instruments. Note that these findings characterize performance-related gaze patterns in the present dataset and should not be interpreted as a causal effect of scan behavior on approach performance in general.

Figure 4 summarizes group-level AOI allocation during the pre-FAF segment quantified via the average proportion of fixations allocated to each AOI and the average proportion of dwell time in each AOI. Across both measures, successful pilots demonstrated a more instrument-centered and operationally efficient scan discipline, emphasizing attitude control, altitude and vertical-speed monitoring, lateral guidance, and power management. Statistically significant differences were found in the successful group, with increased attention to the Alt_VSI (fixation proportion: 0.23 vs. 0.17; dwell time proportion: 0.25 vs. 0.18), RPM (fixation proportion: 0.02 vs. 0.01; dwell time proportion: 0.02 vs. 0.01), and decreased allocation to OTW view (fixation proportion: 0.01 vs. 0.07; dwell time proportion: <0.01 vs. 0.04). Since the Alt_VSI provides information about current altitude and vertical-speed trend, allowing pilots to detect deviations early and anticipate whether the aircraft is moving toward an unstable vertical profile, increased allocation to this AOI suggests stronger vertical-state awareness and more proactive altitude management as the pilots maintain altitude compliance while preparing for transition to the FA segment. Although the RPM received a relatively small proportion of total gaze allocation, successful pilots demonstrated increased attention in this AOI, suggesting power management was integrated into their scan discipline rather than treated as an occasional correction. In an ILS approach, small adjustments in power help stabilize airspeed, descent readiness, and aircraft configuration. As such, even brief but consistent RPM checks will likely support smoother energy management during the pre-FAF segment. Moreover, the successful group showed significantly reduced allocation to the OTW view. In contrast, unsuccessful pilots allocated more visual attention outside, potentially indicating external searching, uncertainty, or a breakdown in instrument scan discipline. The unsuccessful group also showed reduced Alt_VSI allocation, which may indicate weaker vertical-state monitoring, delayed recognition of altitude deviations, or less proactive management of descent readiness before the FAF. The lower RPM allocation in this group further indicates a less complete cross-check, particularly when combined with greater OTW viewing.

Figure 5 shows the top 20 most frequently appearing AOI patterns in each group and sequence support differences across the two groups. In the successful group (Figure 5a), the most frequent pattern was AI → Alt_VSI → AI, which accounted for 4.29% of all pattern occurrences. This was followed by AI → TI_HSI → AI (2.92%), AI → SSI → AI (2.39%), SSI → AI → Alt_VSI (2.33%), and Alt_VSI → AI → Alt_VSI (2.24%). These high-frequency sequences indicate that successful pilots tended to organize their scan around the attitude indicator during the pre-FAF segment, showing a compact but flexible scan structure, with repeated integration of vertical-state information and coordination information.

The unsuccessful group showed a different ordering of top patterns (Figure 5b), with the most frequently exhibited sequence being AI → SSI → AI (2.21%), followed by AI → TI_HSI → AI (2.13%), AI → Alt_VSI → AI (2.00%), SSI → AI → SSI (1.72%), TI_HSI → AI → SSI (1.47%), and SSI → AI → TI_HSI (1.38%). Although the AI remained central in the unsuccessful group, the most frequently exhibited sequences indicate greater relative emphasis on SSI and TI_HSI transitions, suggesting more frequently cycled gaze among attitude, coordination, and lateral/navigation cues, rather than the stronger AI–Alt_VSI coupling observed in the successful group. The results suggest that the unsuccessful group emphasized different instrument relationships, such as more recurrent transitions involving SSI and TI_HSI, which may indicate a scan pattern oriented toward maintaining or correcting lateral alignment and coordination rather than proactively integrating attitude and vertical-state information.

Figure 5c compares sequence-support differences in percentage points (pp) across the groups during the pre-FAF segment. Positive values indicate patterns that were supported more frequently in the successful group, whereas negative values indicate patterns that were supported more frequently in the unsuccessful group. The largest positive difference was observed for SSI → Alt_VSI → AI (+38.3 pp), indicating that this sequence was much more consistently present among successful pilots. This pattern indicates that successful pilots reintegrated coordination checks into altitude/vertical-speed awareness and attitude control. Other positive patterns, such as SSI → AI → SSI (+16.9 pp), AI → SSI → Alt_VSI (+9.7 pp), and Alt_VSI → AI → Alt_VSI (+7.1 pp), similarly indicate that successful pilots were more likely to incorporate SSI and Alt_VSI into short, attitude-centered scan loops. These findings suggest that successful pre-FAF performance is associated with a compact but flexible instrument scan, reflecting an instrument-centered strategy in which attitude control served as a reference point for monitoring altitude/descent readiness, lateral alignment, and aircraft coordination. The most negative differences were observed for SSI → TI_HSI → AI (−22.1 pp), Alt_VSI → SSI → AI (−13.6 pp), AI → TI_HSI → SSI (−13.0 pp), SSI → AI → TI_HSI (−11.0 pp), and TI_HSI → AI → SSI (−11.0 pp). These patterns are dominated by transitions among SSI, TI_HSI, and AI, suggesting greater emphasis on coordination and lateral/navigation monitoring, which may be consistent with a more reactive scan discipline.

### 4.2. Gaze Tendencies During the FA Segment

Figure 6 shows the correlations between gaze measures and FA segment performance that remained statistically significant after B–H false discovery rate correction. The largely negative associations were concentrated predominantly in OTW gaze measures. The strongest negative correlations were observed for the OTW sum of relative degrees (r = −0.733), sum of saccade lengths and sum of saccade amplitudes (r = −0.721 for both), fixation count (r = −0.713), sum of peak saccade velocity (r = −0.694), saccade count (r = −0.692), sum of mean saccade velocity (r = −0.687), sum of saccade durations (r = −0.680), scanpath duration (r = −0.673), sum of fixation durations (r = −0.669), and sum of absolute degrees (r = −0.637). Moderate negative relationships were also observed for OTW convex hull area (r = −0.596), maximum peak saccade velocity (r = −0.544), maximum mean saccade velocity (r = −0.538), maximum saccade amplitude (r = −0.532), maximum saccade length (r = −0.531), the proportion of fixations in OTW (r = −0.522), and maximum relative degree (r = −0.515), as well as the proportion of fixation durations in OTW (r = −0.501). Other non-OTW negative correlations involved ASI convex hull area (r = −0.543), ASI maximum saccade length and amplitude (r = −0.541 for both), and global convex hull area (r = −0.507). Two positive correlations were observed including OTW minimum saccade duration (r = 0.525) and ASI mean relative degree (r = 0.506). Note that the negative relationship between OTW activity and performance should be interpreted specifically in the context of the present study, in which useful external visual references were intentionally limited. Reduced OTW sampling should not be generalized as inherently preferable in other phases of flight or under conditions in which external visual information is operationally necessary.

This pattern suggests that, in the present dataset, higher FA performance was associated with a smaller and less active scan of the OTW region. The ASI associations suggest a more nuanced relationship, where higher-performing pilots showed smaller spatial scan in the ASI region and smaller maximum saccade excursions, whereas the positive ASI mean-relative-degree association may reflect more pronounced directional changes during ASI sampling. The positive association for OTW minimum saccade duration was isolated and should be interpreted cautiously since minimum values are sensitive to individual events.

Figure 7 compares AOI allocation across the groups in the FA segment. Across both fixation count (Figure 7a) and dwell-time (Figure 7b) measures, although the descriptive AOI allocation profiles differed between groups, we did not find statistically significant differences (*p* > 0.05). This result may suggest that performance-related differences may be more evident in how gaze was organized across AOIs rather than in whether the key AOIs were visited. This possibility motivated the subsequent AOI transition and sequence analyses discussed below.

Figure 8 presents the most frequently exhibited AOI sequence patterns during the FA segment for the successful and unsuccessful groups. In this segment, the aircraft had passed the FAF and pilots were required to maintain both localizer and glideslope guidance to DA/DH while also controlling the airspeed, attitude, coordination, and power. Compared with the pre-FAF segment, the FA segment imposed a more tightly coupled lateral–vertical control problem, with less time available to detect and correct deviations.

In the successful group (Figure 8a), the most frequent patterns indicate a compact, instrument-centered scan organized around repeated returns to the attitude indicator and recurrent integration of vertical and lateral guidance cues. Patterns involving AI, Alt_VSI, and TI_HSI are especially meaningful in this segment because they link the core control references needed to maintain pitch/bank attitude, glideslope or vertical-state awareness, and localizer/navigation alignment. During FA, successful pilots appeared to use the AI as an organizing reference while sampling Alt_VSI and TI_HSI in short return-loop structures. This suggests that successful performance was supported by a scan discipline that kept attitude control coupled to both vertical and lateral guidance rather than allowing attention to become isolated on any single instrument. The successful-group patterns can therefore be interpreted as evidence of stable instrument integration under increased task demand. The FA segment requires continuous correction of localizer and glideslope deviations while maintaining airspeed near the target. A scan loop such as AI → Alt_VSI → AI indicates that vertical-state information was being brought back into the attitude-control reference, while AI → TI_HSI → AI indicates that lateral/navigation information was being integrated into the same attitude-centered framework. When such patterns are frequent, they may suggest that the pilot is not merely checking instruments independently but using them as part of a coordinated control loop.

In contrast, the unsuccessful group’s (Figure 8b) most frequent patterns suggest a less efficiently integrated scan structure. Although the AI remained present in many recurrent sequences, unsuccessful patterns tended to show greater coupling among instruments associated with lateral correction, coordination, or compensatory monitoring. In the FA segment, such a scan structure may indicate that pilots were repeatedly checking lateral alignment, coordination, or airspeed after deviations had already developed.

The sequence-support differences (Figure 8c) provide a complementary group-level comparison. Patterns with positive differences can be interpreted as scan structures that are more characteristic of successful FA performance, whereas negative differences identify patterns more characteristic of unsuccessful FA performance. The strongest positive differences were observed for AI → SSI → AI (+13.0 pp) and SSI → Alt_VSI → SSI (+11.0 pp), with smaller positive differences for AI → Alt_VSI → TI_HSI (+1.3 pp) and SSI → AI → SSI (+1.3 pp). These successful-group patterns suggest that higher-performing pilots retained short, compact scan loops involving attitude, coordination, vertical-state, and lateral-guidance information. The largest negative difference was AI → SSI → TI_HSI (−35.7 pp), followed by Alt_VSI → SSI → AI (−19.5 pp), SSI → AI → TI_HSI (−19.5 pp), AI → TI_HSI → SSI (−16.9 pp), and Alt_VSI → SSI → Alt_VSI (−16.9 pp). These patterns are dominated by transitions among AI, SSI, TI_HSI, and Alt_VSI. In the FA segment, this may indicate a scan structure oriented toward correcting or monitoring deviations in coordination and lateral/navigation state. These findings suggest that unsuccessful pilots may have used relevant instruments in a less effective sequence structure. For example, the strong unsuccessful-group support for AI → SSI → TI_HSI and SSI → AI → TI_HSI suggests repeated movement from attitude and coordination cues toward lateral/navigation monitoring, possibly reflecting a reactive correction cycle, especially if pilots were repeatedly checking alignment or coordination after deviations had already developed.

### 4.3. Gaze Tendencies of the Overall ILS Approach

Figure 9 presents the top 20 patterns exhibited by each group during the overall approach. Nodes represent AOIs, where each column represents the ordinal position of an AOI within a sequence, and the width of each flow reflects the frequency of that transition. Position labels (i.e., Position 1, Position 2, and Position 3) indicate the first, second, and third AOI in a scan sequence. The number shown below an AOI label is the summed pattern frequency for that AOI at that ordinal position, and the link width is proportional to the summed frequency of transitions between adjacent AOI positions across all top 20 patterns. As such, wider flows indicate more frequently recurring scan transitions.

In the successful group (Figure 9a), the most frequent scan sequence was AI → Alt_VSI → AI (frequency: 1054), followed by AI → TI_HSI → AI (frequency: 754). These two dominant sequences indicate that throughout the approach, successful pilots repeatedly returned to the attitude indicator after checking vertical-state and lateral/navigation information. The successful group also showed frequent patterns involving SSI and AI, including AI → SSI → AI and SSI → AI → Alt_VSI (both with frequency: 535), indicating that coordination checks were commonly reintegrated into attitude and vertical-state monitoring. The sequence Alt_VSI → AI → Alt_VSI (frequency: 531) further indicates repeated cross-checking between attitude and vertical-state cues. Overall, the scan patterns in the successful group indicate a compact but flexible scan architecture, with recurrent loops linking attitude, vertical state, lateral/navigation guidance, and coordination. Patterns involving the undefined area were limited primarily to brief, SSI-centered loops, which may reflect brief gaze excursions near the boundary of the SSI AOI rather than a generalized breakdown of instrument scanning. In other words, gaze in the undefined area appeared relatively constrained and patterned, suggesting that when successful pilots’ gaze entered the undefined area, it was typically embedded within a local transition structure around the SSI rather than distributed broadly across the scan sequence.

The unsuccessful group (Figure 9b) showed a different organization of scan patterns. The most frequent patterns were AI → SSI → AI and AI → TI_HSI → AI (both with frequency: 366), followed by SSI → AI → SSI (frequency: 295) and AI → Alt_VSI → AI (frequency: 281). These patterns indicate scans that were more strongly oriented toward attitude–coordination and attitude–lateral/navigation loops with weaker coupling between attitude control and vertical-state monitoring. The unsuccessful group also showed more frequent and more diverse transitions into and out of the undefined area (with over 60% more summed transitions involving the undefined area compared to the successful group). This finding indicates that the undefined area was not confined to a single local AOI boundary or one instrument-adjacent loop but, rather, across broadly distributed transitions. Such a pattern suggests a less compact scan structure, in which gaze more often moved away from task-relevant AOIs. As such, frequent transitions through the undefined area may indicate inefficient visual routing, unstable scan anchoring, or brief losses of alignment between gaze behavior and task-relevant instrument regions.

ASI-related scan patterns further differentiate the groups. The successful group exhibited the pattern Alt_VSI → AI → ASI, whereas this pattern was not present among the most frequent in the unsuccessful group. This distinction suggests that effective approach control requires pilots to manage the descent path and airspeed together. A vertical deviation often requires interpretation through pitch attitude and may also require airspeed or power correction. Therefore, a scan sequence such as Alt_VSI → AI → ASI may reflect an anticipatory control strategy, whereby the pilot checks vertical state, verifies or adjusts attitude, and then confirms whether the energy state remains appropriate. The absence of this pattern in the unsuccessful group may indicate that vertical and energy information were monitored in a more fragmented manner, reducing the pilot’s ability to detect and correct coupled deviations early. These findings further extend the segment-level analysis by highlighting that successful performance was associated not simply with more instrument viewing but with more functionally integrated scan organization.

## 5. Implications for Pilot Training and Flightdeck Design

The AOI sequences observed in this study provide descriptive behavioral patterns showing recurrences within the present dataset; while they should not be interpreted as validated markers of optimal instrument scanning, they nonetheless motivate the approach to potentially evaluate scan discipline as a phase-sensitive and sequence-dependent behavior. Evidentially, a pilot may visit the correct AOIs yet still show degraded scan discipline if those visits are poorly sequenced, if attention becomes trapped in lateral or airspeed correction loops, or if gaze repeatedly leaves task-relevant instrument regions. Based on the observed group differences, we propose a set of segment-specific gaze metrics that may support simulator debriefing and scan discipline assessment. These metrics are intended to complement conventional performance measures rather than replacing them as standalone pass/fail indicators. Specifically, allocation-based metrics may be helpful to quantify whether visual attention was directed toward task-relevant AOIs, while sequence-based metrics may be useful to quantify whether those AOIs were organized into operationally meaningful scan patterns. As such, the proposed metrics should be regarded as candidate gaze-based indicators derived from the present dataset rather than validated assessment-ready metrics. Their reliability, discriminative validity, and prospective association with approach performance require further evaluation in independent samples before operational use.

### 5.1. Proposed Metrics for the Pre-FAF Segment

The proposed metrics for the pre-FAF segment, shown in Table 3, aim to capture three elements of scan discipline, including suppression of low-value external sampling, integration of vertical-state and power information, and avoidance of excessive lateral/coordination dominance. The OTW suppression index and instrument-to-OTW allocation ratio quantify whether the pilot remains visually committed to the instrument panel during a phase in which external cues provide limited operational value. Additionally, the sequence-based metrics provide complementary information about how instrument checks are organized. The AI-centered cross-check rate evaluates whether attitude remains the organizing reference for the scan, whereas the vertical-integration rate and coordination-to-vertical recovery index evaluate whether altitude and vertical-speed information are reintegrated after coordination or lateral checks. These metrics may be useful for distinguishing a balanced pre-FAF cross-check from a reactive scan dominated by lateral correction. The lateral-coordination dominance index provides a risk-oriented measure of this tendency, whereby elevated values may indicate that the pilot is repeatedly cycling through SSI and TI_HSI without sufficient return to Alt_VSI or AI. The pre-FAF task-relevant scan balance score summarizes whether gaze is weighted toward instruments that are most relevant to localizer capture, altitude compliance, or power management.

### 5.2. Proposed Metrics for the FA Segment

The proposed metrics for the FA segment, outlined in Table 4, aim to determine whether pilots maintain an integrated attitude-centered cross-check under an increased workload. The compact scan index summarizes whether gaze remains spatially economical, whereas the OTW activity penalty captures the degree to which attention is drawn outside during a phase where instrument integration is critical. The guidance–integration allocation ratio evaluates whether gaze is weighted toward the core guidance instruments (AI, Alt_VSI, and TI_HSI) or toward compensatory regions. While ASI monitoring is necessary during this segment, excessive ASI fixation, scanpath duration, or spatial dispersion may indicate speed fixation or reactive energy correction. The ASI over-monitoring index aims to evaluate this. The sequence pattern metrics aim to identify whether pilots recover quickly from potentially reactive checks. The AI return-loop rate evaluates whether pilots return to attitude after checking other AOIs, and the coupled guidance–integration rate assesses whether attitude, vertical guidance, and lateral/navigation information are linked in recurrent scan sequences. Elevated values on the ASI fixation-loop index, lateral-correction loop index, or undefined area interruption index may indicate that the scan is becoming reactive, fragmented, or poorly anchored.

### 5.3. Proposed Metrics for the Overall Approach

In addition to segment-specific metrics, global and composite metrics may be useful for summarizing scan organization across the full ILS approach. These measures are not intended to duplicate the pre-FAF and FA segment-specific metrics, but rather to capture higher-level properties of scan behavior that emerge across both segments. Whereas segment-specific metrics diagnose whether gaze behavior was appropriate for a particular phase of flight, the overall approach metrics (Table 5) summarize whether the pilot maintained a coherent scan discipline across the maneuver and whether the scan adapted as task demands changed. These global measures may help instructors identify whether a pilot’s scan is consistently organized, appropriately adaptive, vertically and energetically integrated, or fragmented across phases of flight.

One useful class of global metrics concerns similarity to successful scan organization. An overall scan similarity score could compare an individual pilot’s full-approach sequence-support profile with the established reference profile (e.g., observed among successful pilots). This may provide a single interpretable index of how closely a pilot’s scan organization resembles the successful group, while still allowing instructors to inspect the underlying sequence patterns when needed. Another class of metrics concerns functional integration across control dimensions. The overall vertical-energy coupling score may capture whether pilots connect descent-path monitoring, attitude control, and airspeed management into a single functional loop. It differs from segment-specific ASI or Alt_VSI measures, as it reflects whether vertical and energy-state information are integrated across the overall approach rather than monitored as separate tasks. A third class of metrics concerns scan stability and fragmentation. A localized-versus-distributed undefined area ratio could separate likely AOI-boundary effects from broader scan disruption. Similarly, a full-approach fragmentation index, based on the number of unique recurrent patterns relative to the total pattern frequency or on transition entropy, could indicate whether the pilot’s scan was stable and repeatable or diffuse and poorly organized. Finally, a phase-transition adaptation score could quantify how gaze behavior changes from the pre-FAF segment to the FA segment. This metric would capture whether the pilot appropriately shifts scan emphasis as task demands change. For example, an effective transition may involve maintaining AI-Alt_VSI coupling while increasing integration of lateral and vertical guidance cues during FA, without increasing OTW, undefined area, or ASI-fixation patterns.

### 5.4. Implications for Training Interface and Flightdeck Design

The proposed metrics can also be translated into practical simulator-training and flightdeck interface features. While not intended to replace existing instrument procedures or certified avionics displays, they may be useful to inform training displays, simulator debriefing tools, and future human–machine interface concepts that support more effective instrument-scan discipline. The central design objective is to help pilots and instructors see whether gaze behavior is compact, attitude-centered, vertically integrated, and appropriately adaptive across segments, or reactive, fragmented, externally interrupted, or poorly anchored.

As an example, in the context of a segment-specific scan dashboard for simulator debriefing, after an ILS scenario, the interface could display separate pre-FAF, FA, and overall-approach panels, where each panel may report the proposed metrics outlined in Table 3, Table 4 and Table 5. If a pilot showed a low OTW suppression index and low Alt_VSI monitoring proportion before the FAF, the debriefing display could flag, “Pre-FAF scan was externally biased; altitude and vertical-speed monitoring were underrepresented”. Similarly, if SSI and TI_HSI dominated sequence patterns before the FAF, the interface could report, “Scan showed lateral/coordination dominance with limited reintegration of vertical state”. Such feedback would directly connect gaze behavior to the operational demands of localizer capture, altitude compliance, and descent preparation.

During debriefing, AOI sequence replay visualizing gaze transitions as a time-ordered scanpath may also be useful, as it complements existing analytical tools such as heat maps, in which a replay could display a pilot’s AOI sequence alongside the flight path, localizer deviation, glideslope deviation, and airspeed trace. During debriefing, an instructor could move to a point in the replay where the aircraft deviated from the glideslope and show that the pilot’s gaze was cycling through SSI and TI_HSI without returning to Alt_VSI. This would help distinguish a control-performance error from the visual strategy that preceded it.

Likewise, it may be useful to display an undefined area diagnostic overlay that separates “localized undefined area involvement” from “distributed undefined area involvement”, rather than treating all undefined gaze samples as equivalent. This distinction may prevent overinterpretation of minor boundary effects while still identifying scan instability when undefined area transitions are widespread.

Similarly, a debriefing interface could display phase-transition adaptation showing how a pilot’s scan metrics changed across the FAF. For example, the display could compare pre-FAF and FA values for AI return-loop rate, vertical-integration rate, OTW activity, ASI over-monitoring, and undefined area interruption. A stable and adaptive scan may show continued AI-Alt_VSI coupling while increasing integration of lateral and vertical guidance during FA. A less effective scan might show increased ASI looping, greater undefined area dispersion, or persistent lateral-coordination dominance after the FAF. This would allow instructors to evaluate whether the pilot adapted scan behavior to the changing control demands of the approach.

Potential considerations for future flightdeck design may include supporting not only the visibility of individual instruments, but also the functional relationships among them. For example, a low-salience halo may be displayed to encourage vertical verification as the next scan element after an attitude update; or a transient energy-state cue to briefly relate airspeed trend to pitch and descent rate. Any such cueing should be transient, low-salience, and context-aware, appearing only when operationally meaningful. Cueing should not introduce sustained clutter, additional cognitive steps, nuisance alerts, or mode confusion. The goal is not to add more information, but to make the relationships among existing information sources easier to perceive at a moment that they are operationally relevant. The envisioned future flightdeck is one that can support expert-like scan discipline by reinforcing the same functional loops observed in successful approaches, though these potential considerations should be evaluated carefully in simulator and operationally realistic settings to ensure that they improve scan efficiency without disrupting pilot autonomy, increasing workload, or encouraging overreliance on visual cueing.

## 6. Conclusions and Future Work

This study compares gaze behavior and scan discipline between successful and unsuccessful pilots during simulated ILS approaches in a specific scenario, adding to the existing body of knowledge on how eye tracking can support better understanding of scan discipline, and contributing to enhanced pilot training, assessment, and flightdeck-interface research. The findings show that successful approaches were characterized by a more compact, instrument-centered, and functionally integrated scan structure. Additionally, performance-relevant gaze behavior is defined not only by where pilots looked, but also by how instrument checks were sequenced over time, indicating that effective ILS scan discipline is phase-sensitive and sequence-dependent. The proposed assessment metrics provide one potential framework for translating gaze data into interpretable training and design measures, while noting several limitations below.

First, this study was conducted in a simulated environment, providing a condition to isolate gaze–performance relationships but does not reproduce the full sensory and operational environment of an aircraft or full-motion simulator. As such, the results should be generalized cautiously to other aircraft, avionics layouts, weather conditions, and operational contexts, since the magnitude and organization of visual scanning observed here may differ under operational flight conditions, and external validation in higher-fidelity simulation and actual flight is required. Second, the approach scenario was intentionally controlled, with fixed weather and a single attempt per participant, which improved experimental consistency but limited the range of operational variability. Third, the sample included pilots with varied certification levels and flight experience, but the number of participants limits the extent to which subgroup effects, such as instrument currency, total flight time, or recent approach experience, can be separately modeled. Additionally, undefined area patterns should be interpreted with care since they may reflect AOI-boundary effects, calibration uncertainty, or transitional gaze movements as well as less stable scan anchoring. The observed gaze patterns were associated with successful approach performance in this study but should not be interpreted as evidence that those gaze patterns caused better performance. Furthermore, the proposed assessment metrics were derived from gaze-performance relationships observed in the present sample and should not be regarded as validated diagnostic measures. Future studies should evaluate their reliability, discriminative validity, and predictive performance in larger independent samples.

Future work can extend this study in several directions. For instance, the proposed gaze metrics should be validated across different levels of pilot experience, instrument currency, and aircraft familiarity. Given that each participant completed one experimental approach, the present data characterize scan behavior during that specific performance episode. Future studies may obtain repeated approaches under multiple conditions to estimate within-participant stability and separate systematic scan behavior from trial-specific variation. In addition, future studies could examine whether the identified scan patterns can predict approach performance prospectively, rather than distinguishing performance after the fact. On a related topic, the sequence analyses characterize recurrent ordering of AOI visits but do not establish whether a given pattern preceded or followed a flightpath or airspeed deviation, which may be useful in synchronized performance–gaze analyses. Moreover, additional approach types and environmental conditions should be evaluated to determine whether the same scan-discipline markers generalize beyond the present scenario. Furthermore, future studies could evaluate whether sequence-based eye-tracking feedback in training does improve subsequent approach performance, to compare against conventional instructor feedback in learning and retention. Likewise, future interface-design research could examine transient, low-salience, and context-aware cueing concepts that reinforce expert-like cross-check patterns without adding clutter or mode confusion.

## Figures and Tables

**Figure 1:**
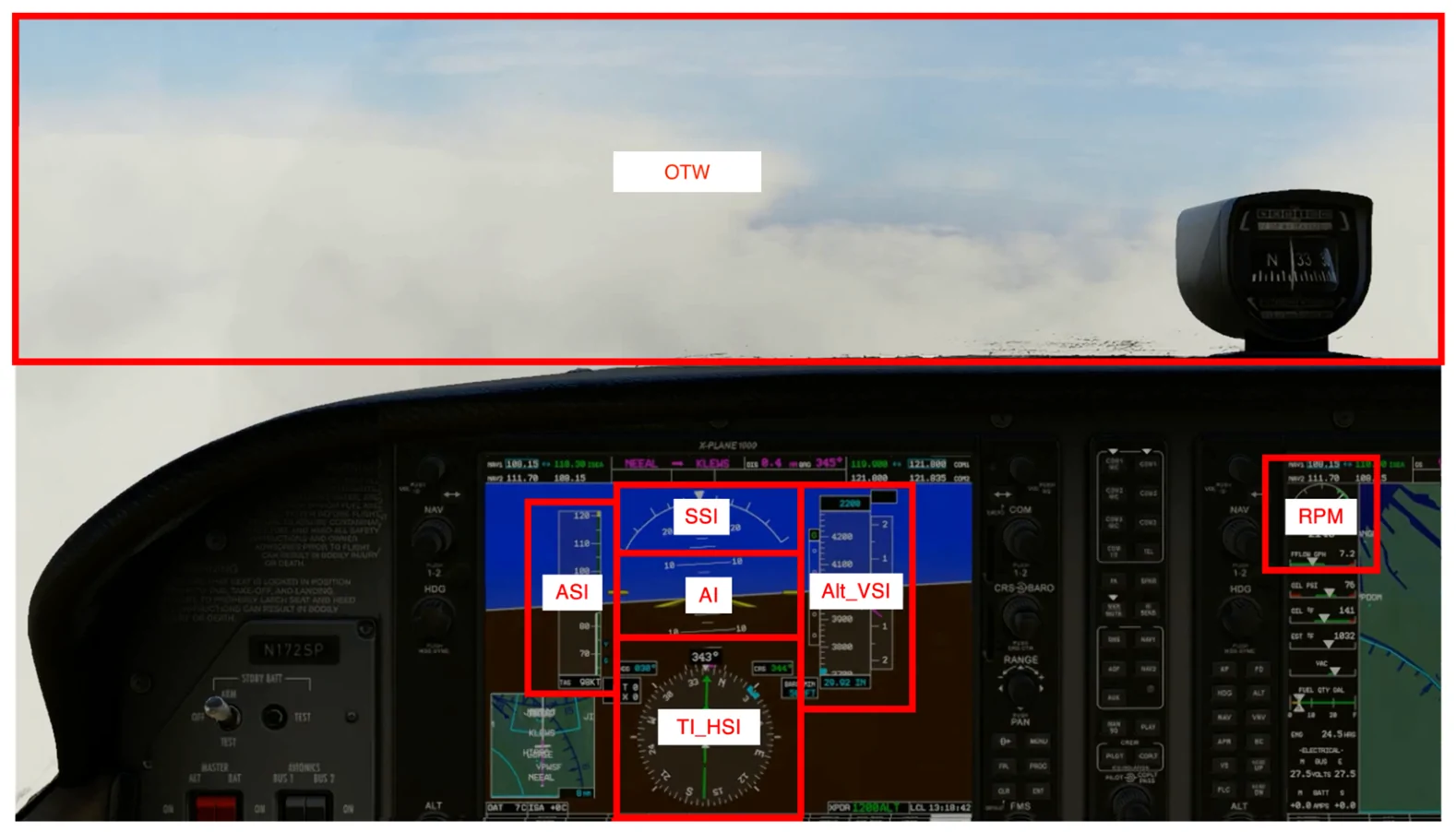
Participants’ view of the simulated flightdeck of a Cessna 172 equipped with the G1000 avionics. The red outlines mark the areas of interest (AOIs), consisting of the out-the-window (OTW) view and the primary flight instruments: slip–skid indicator (SSI), airspeed indicator (ASI), attitude indicator (AI), altimeter/vertical speed indicator (Alt_VSI), turn indicator/horizontal situation indicator (TI_HSI), and tachometer (RPM). Undefined area refers to valid gaze recordings that fell outside the seven task-specific AOIs as outlined, while noting that such recordings can arise from genuine gaze allocation outside the predefined AOIs, transitions between them, proximity to AOI boundaries, or calibration uncertainty.

**Figure 2:**
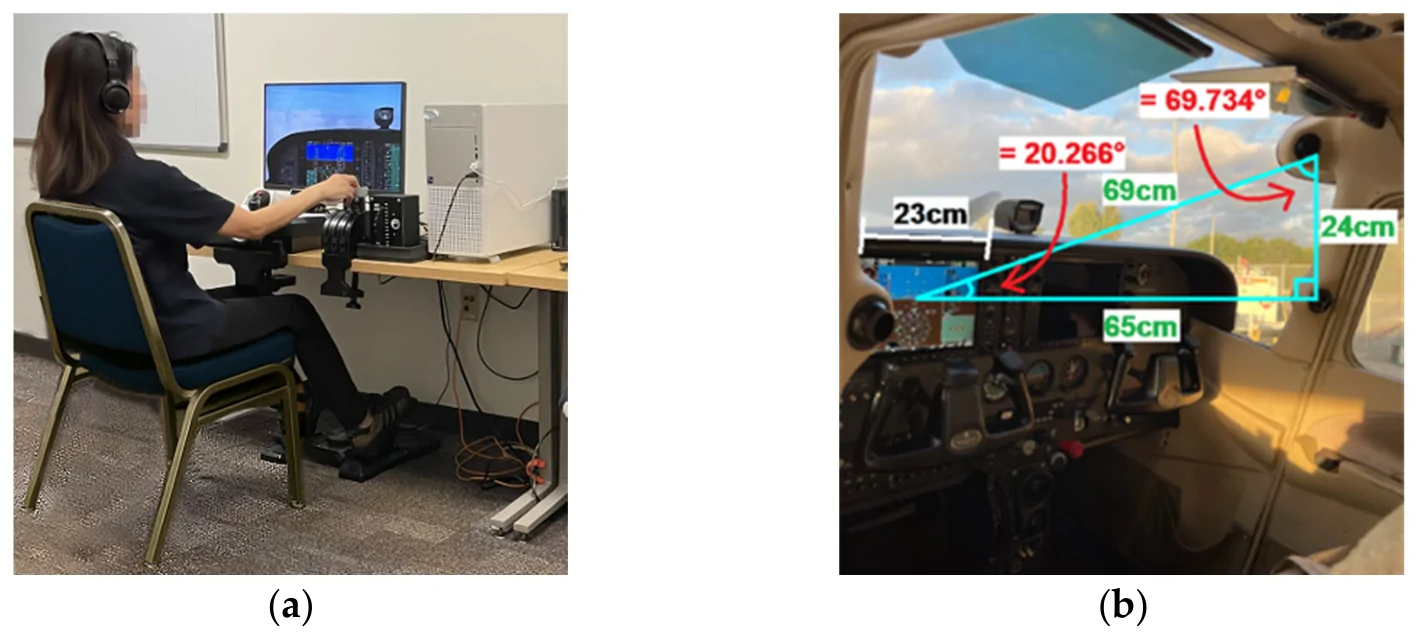
Experimental setup for the simulated ILS task. (**a**) Physical configuration of the study; note that the flightdeck display and perspective were fixed for all participants and remained constant throughout the study. (**b**) Angle of incidence in an actual Cessna 172 with G1000 avionics.

**Figure 3:**
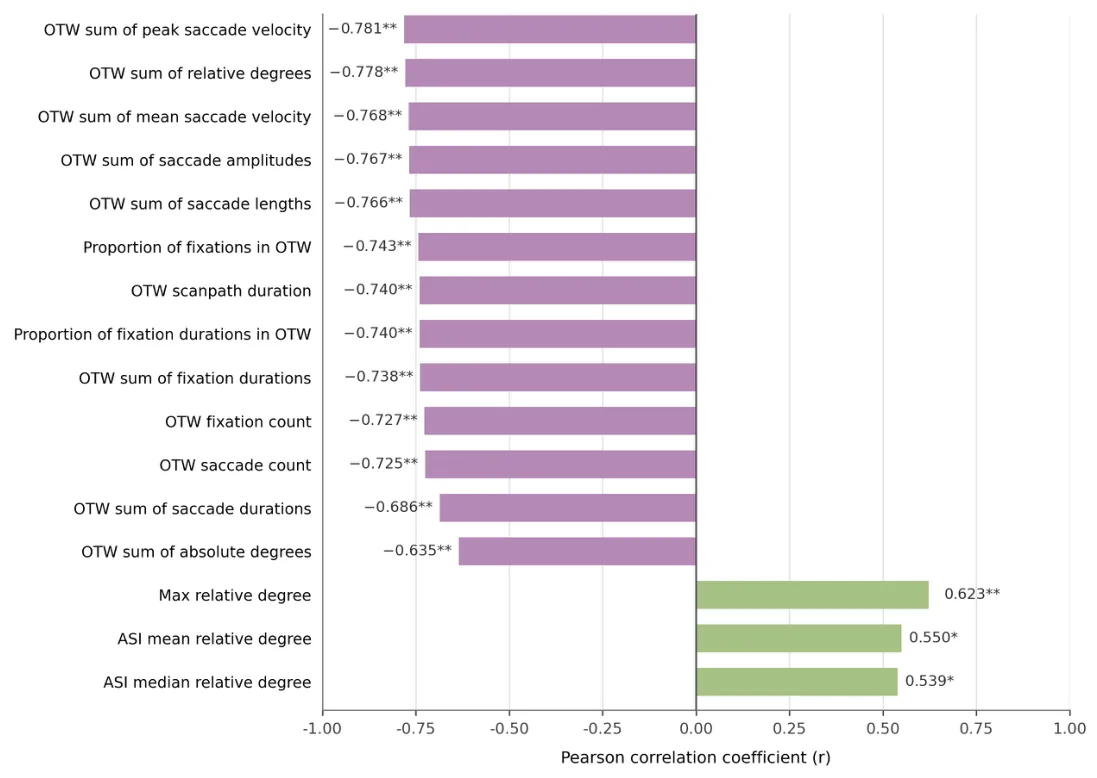
Strong (1.0 ≥ |r| ≥ 0.6) to moderate (0.6 > |r| ≥ 0.4) Pearson correlations (r) observed between gaze measures and pre-FAF segment scores that remained statistically significant following B–H false discovery rate correction across 466 tests in the analysis family (* q < 0.01, ** q < 0.001). When a gaze measure makes no mention of the AOI, it refers to a global value captured for the entire task screen.

**Figure 4:**
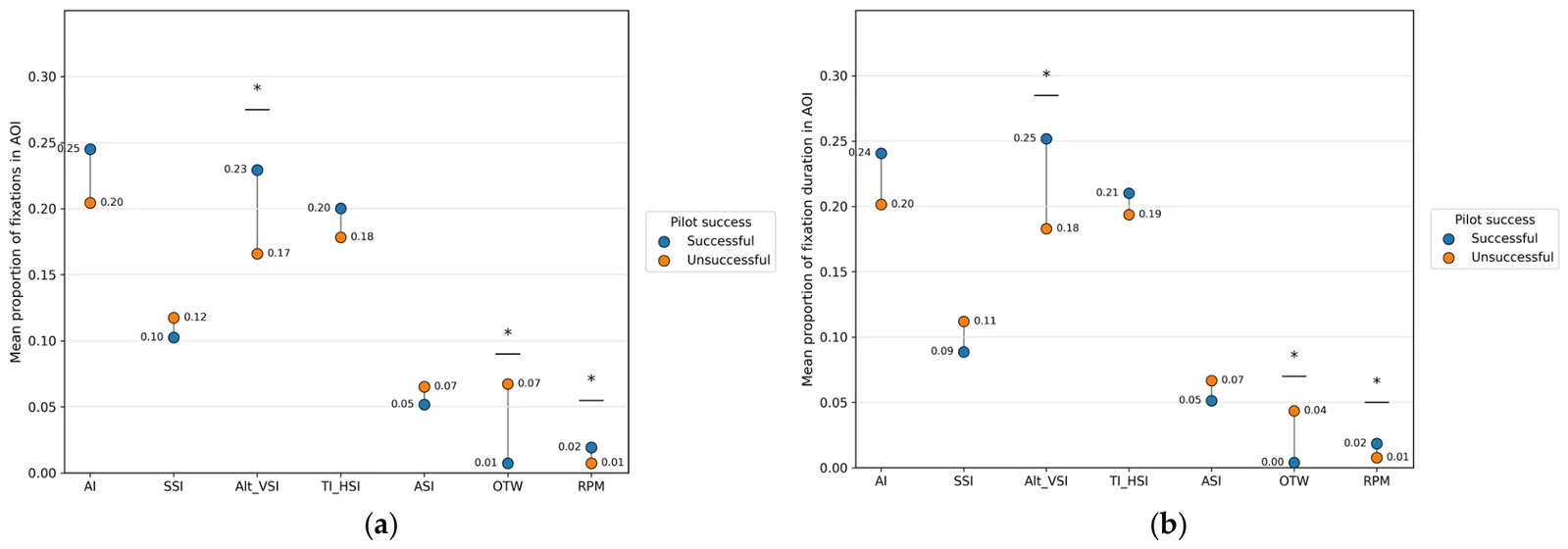
Pre-FAF segment gaze tendencies across AOIs by group (* *p* < 0.05). (**a**) Mean proportion of fixations in AOI; (**b**) Mean proportion of dwell time in AOI. Effect size for the mean proportion of fixations in AI: *g* = 0.42, SSI: *g* = −0.21, Alt_VSI: *g* = 0.75, TI-HSI: *g* = 0.19, ASI: *g* = −0.48, OTW: *g* = −1.00, RPM: *g* = 0.66; and the mean proportion of dwell time in AI: *g* = 0.35, SSI: *g* = −0.30, Alt_VSI: *g* = 0.69, TI-HSI: *g* = 0.12, ASI: *g* = −0.47, OTW: *g* = −0.99, RPM: *g* = 0.61, where positive *g* indicates greater allocation in the successful group and negative *g* indicates greater allocation in the unsuccessful group.

**Figure 5:**
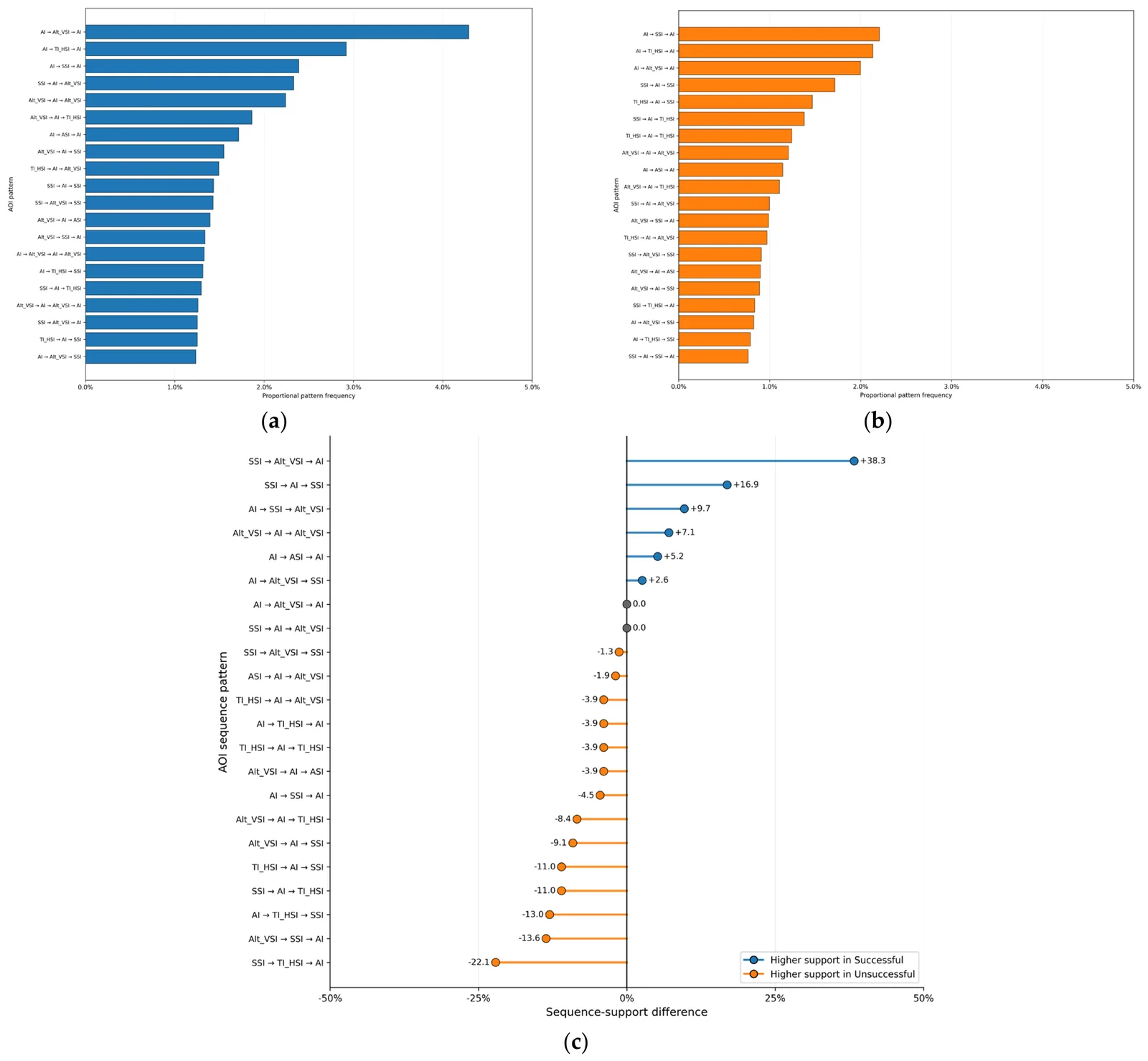
Pre-FAF segment top 20 AOI pattern frequencies and sequence support by group. (**a**) Most frequent patterns in the successful group; (**b**) Most frequent patterns in the unsuccessful group; (**c**) Percentage point (pp) differences of sequence support between groups.

**Figure 6:**
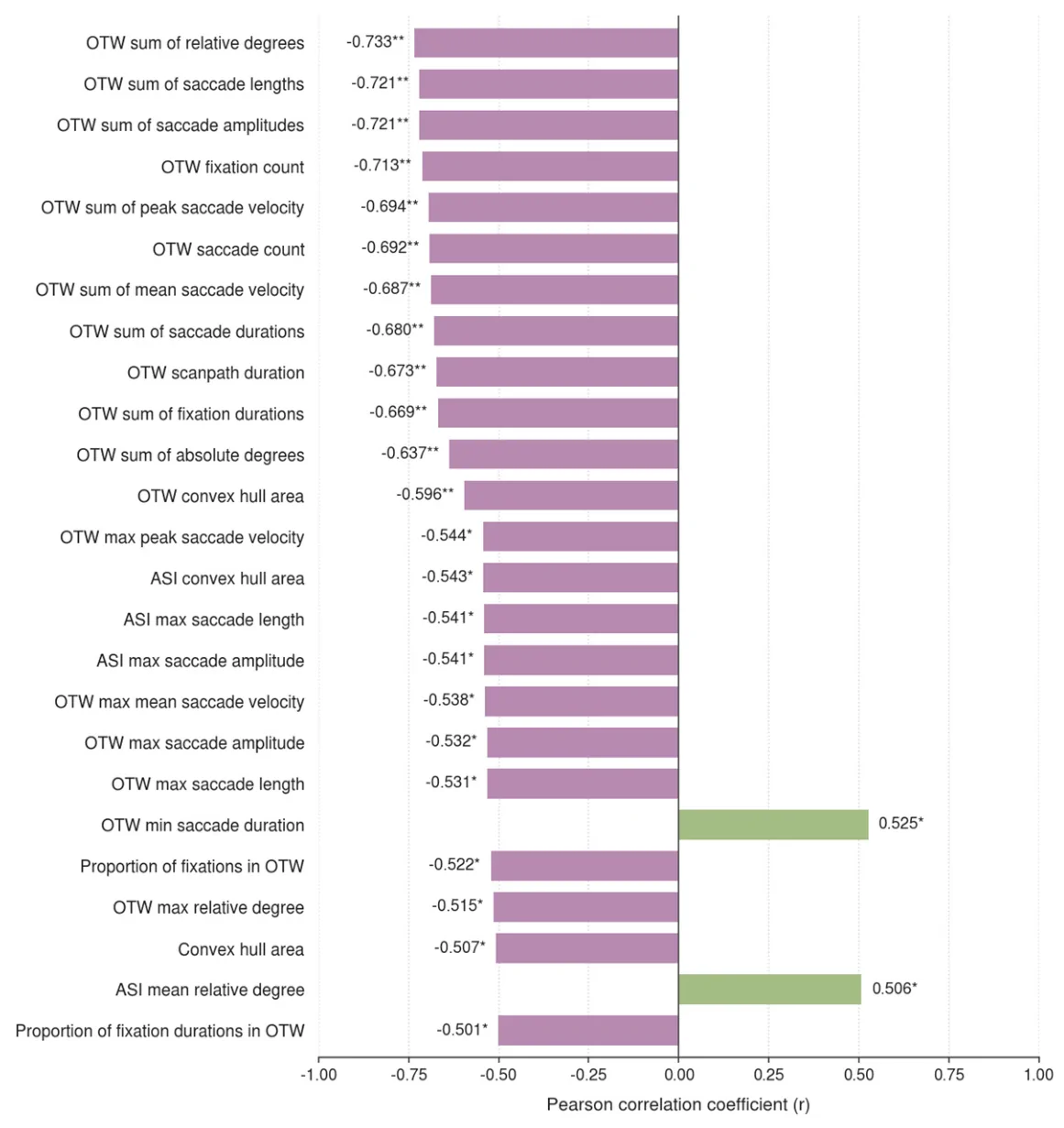
Strong (1.0 ≥ |r| ≥ 0.6) to moderate (0.6 > |r| ≥ 0.4) Pearson correlations (r) observed between gaze measures and FA segment scores that remained statistically significant following B–H false discovery rate correction across 466 tests in the analysis family (* q < 0.01, ** q < 0.001). When a gaze measure makes no mention of the AOI, it refers to a global value captured for the entire task screen.

**Figure 7:**
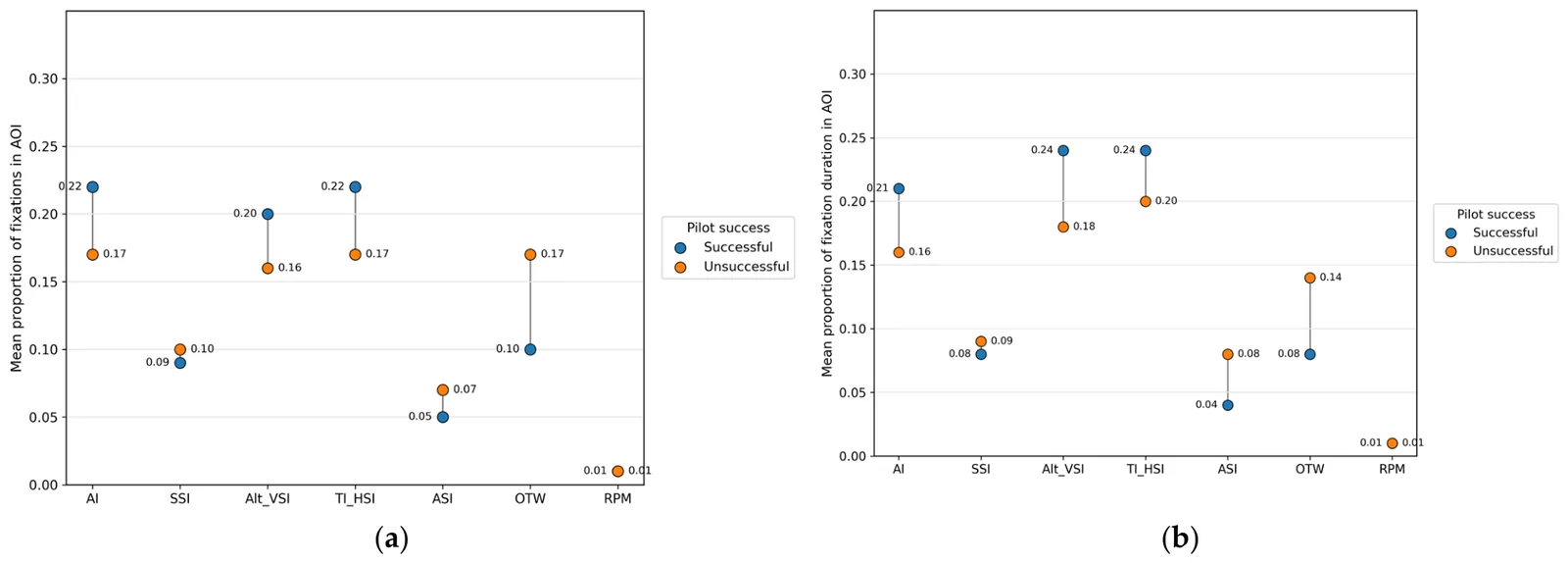
FA segment gaze tendencies across AOIs by group (*p* > 0.05). (**a**) Mean proportion of fixations in AOI and (**b**) mean proportion of dwell time in AOI. Effect size for the mean proportion of fixations in AI: *g* = 0.49, SSI: *g* = −0.09, Alt_VSI: *g* = 0.37, TI-HSI: *g* = 0.37, ASI: *g* = −0.55, OTW: *g* = −0.77, and RPM: *g* = 0.26, and the mean proportion of dwell time in AI: *g* = 0.46, SSI: *g* = −0.15, Alt_VSI: *g* = 0.41, TI-HSI: *g* = 0.24, ASI: *g* = −0.68, OTW: *g* = −0.75, and RPM: *g* = 0.17, where positive *g* indicates greater allocation in the successful group and negative *g* indicates greater allocation in the unsuccessful group.

**Figure 8:**
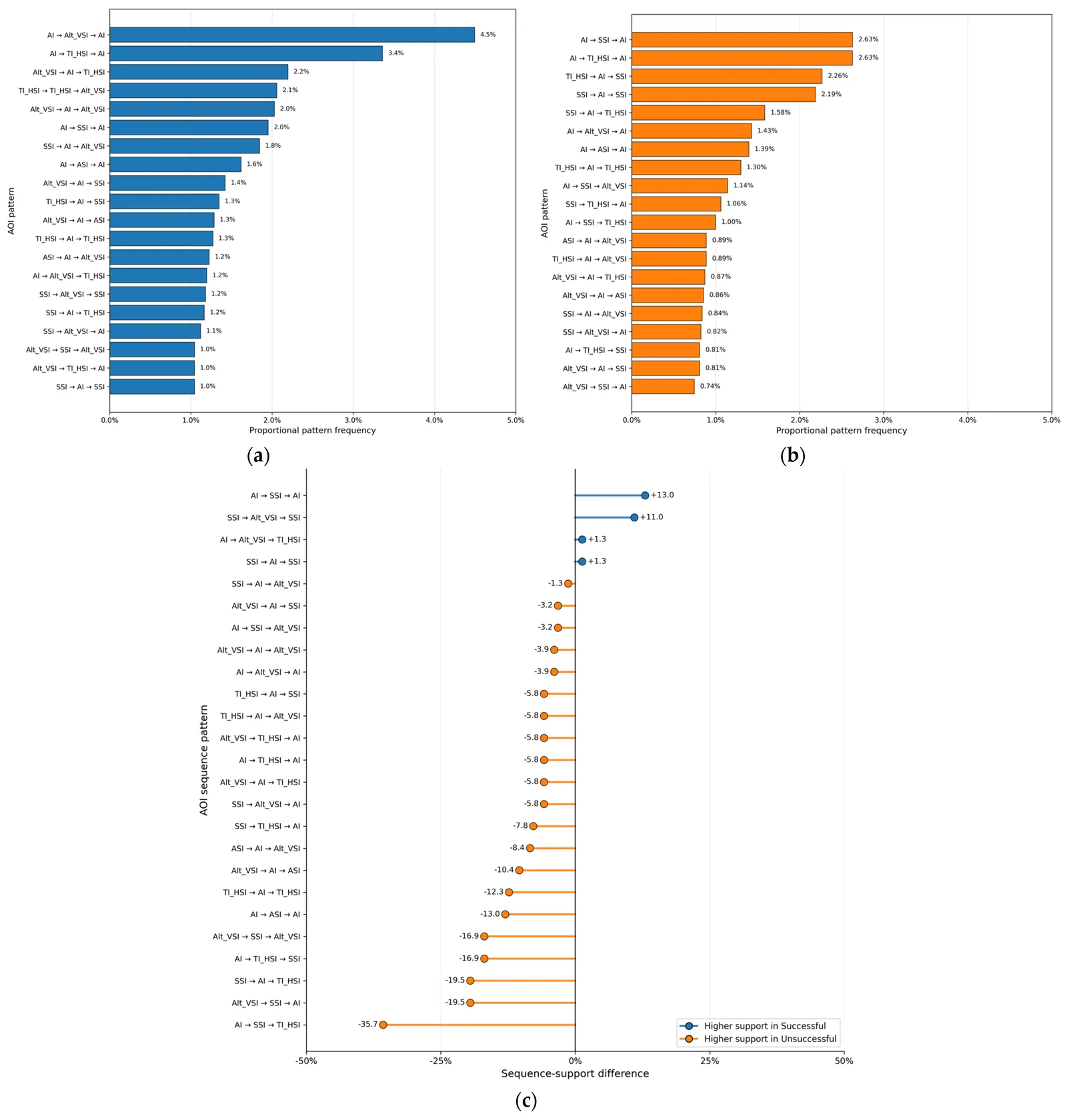
FA segment top 20 AOI pattern frequencies and sequence support by group. (**a**) Most frequent patterns in the successful group; (**b**) Most frequent patterns in the unsuccessful group; (**c**) Percentage point (pp) differences of sequence support between groups.

**Figure 9:**
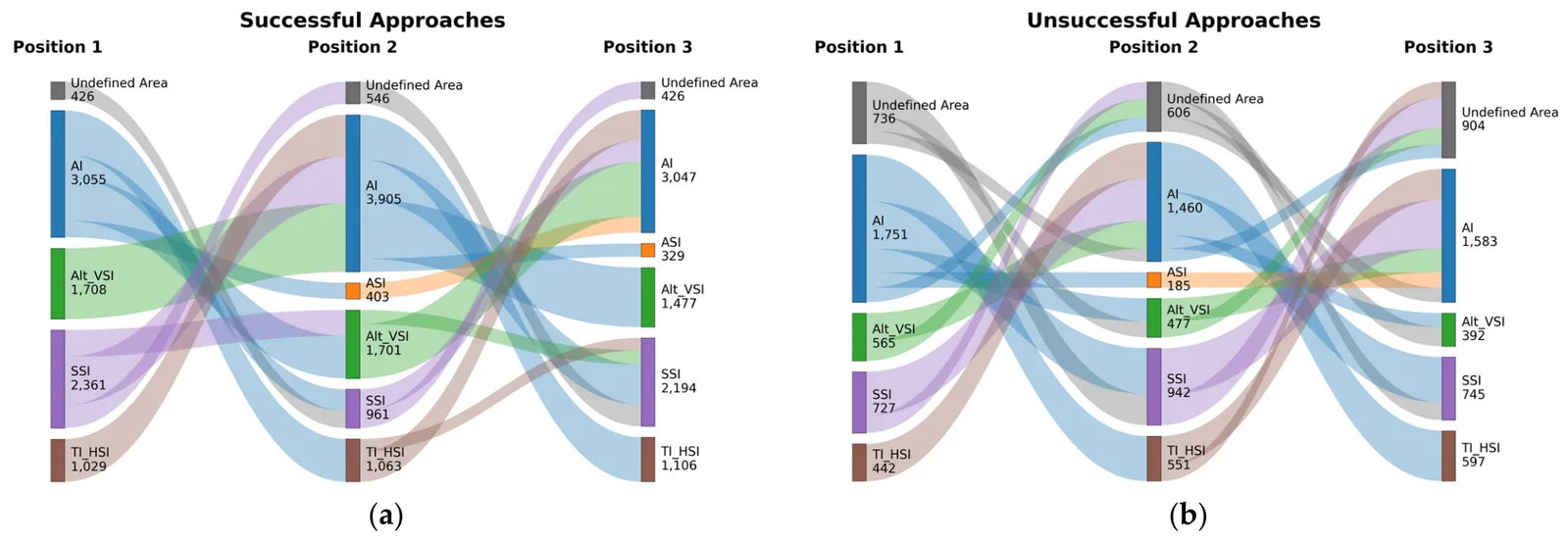
Top 20 AOI sequence patterns by group exhibited in the overall approach including all segments. (**a**) Patterns in the successful group; (**b**) Patterns in the unsuccessful group.

**Table 1:** Gaze measures generated for each pilot.

Feature	Descriptive Measures Derived
Fixations	Count; Duration*; Convex Hull.
Saccades	Count; Magnitude*; Duration*; Amplitude*, Velocity*; Peak Velocity*; Relative and Absolute Direction*.
Blinks	Blink Rate
Pupil	Left Eye Size and Dilation; Right Eye Size and Dilation; Average Pupil Size and Dilation across Both Eyes.
Combined	Fixation-to-saccade Ratio; Scanpath Duration

* Sum, mean, median, standard deviation, minimum, and maximum values were also generated.

**Table 2:** AOI-specific gaze measures generated for each pilot.

Measure	Definition
Stationary entropy	The distribution of a person’s gaze across AOIs
Transition entropy	The extent to which a person has transitioned between the different AOIs
Proportion of fixations within AOI	The percentage of fixation counts that occurred in an AOI
Proportion of fixation duration within AOI	The percentage of fixation durations that occurred in an AOI
AOI pair	Identifies the two AOIs in which a gaze transitions from the first to the second in sequence
Transition count	The number of transitions that occurred for an AOI pair
Proportion of transition including self-transitions	The proportion of total transitions that occurred between AOI pairs, inclusive of self-transitions
Proportion of transition excluding self-transitions	The proportion of total transitions that occurred between AOI pairs, exclusive of self-transitions
Pattern of AOI	A string of characters representing a sequence of AOIs, either expanded or collapsed
Pattern frequency	The number of times a pattern appears in a person’s sequence of AOIs visited, either expanded or collapsed
Sequence support	The proportion of sequences in a user group where the pattern appears, relative to the total number of sequences identified for that group, either expanded or collapsed

**Table 3:** Proposed assessment metrics for the pre-FAF segment.

Metric	Definition	Training Interpretation
OTW suppression index	1—OTW fixation-duration proportion	Higher values indicate better suppression of low-value external viewing. Lower values may indicate external searching or weak instrument reliance
Instrument-to-OTW allocation ratio	(AI + Alt_VSI + ASI + RPM + SSI + TI_HSI fixation duration)/OTW fixation duration	Higher values indicate a more instrument-centered scan. Lower values may indicate excessive external visual sampling
Vertical-power scan index	Alt_VSI fixation-duration proportion + RPM fixation-duration proportion	Captures combined monitoring of vertical state and power management during localizer capture and descent preparation
AI-centered cross-check rate	Proportion of sequence patterns containing AI as the first, middle, or return AOI, e.g., AI → Alt_VSI → AI, AI → TI_HSI → AI, and AI → SSI → AI	Indicates whether the pilot uses the attitude indicator as the organizing reference for the pre-FAF scan
Coordination-to-vertical recovery index	Proportion of SSI-involving patterns that transition to Alt_VSI or AI within the next one or two AOIs	Indicates whether coordination checks are reintegrated into vertical-state and attitude monitoring
Lateral-coordination dominance index	Proportion of sequence patterns dominated by SSI and TI_HSI transitions, such as AI → SSI → TI_HSI, SSI → TI_HSI → AI, and SSI → AI → TI_HSI	Elevated values may indicate a reactive scan focused on lateral alignment or coordination rather than balanced instrument integration
Pre-FAF task-relevant scan balance score	(AI + Alt_VSI + TI_HSI + RPM) − (OTW + outside task-relevant AOI area) using fixation-duration proportions	Positive values indicate gaze weighted toward instruments most relevant to localizer capture, altitude compliance, and power management

**Table 4:** Proposed assessment metrics for the FA segment.

Metric	Definition	Training Interpretation
Compact scan index	1—normalized convex hull area	Higher values indicate a compact scan. Lower values may indicate dispersed visual search or unstable scan control
OTW activity penalty	OTW fixation and duration proportion	Higher values indicate greater engagement with the outside scene
Guidance–integration allocation ratio	(AI + Alt_VSI + TI_HSI fixation duration)/(ASI + SSI + OTW + Undefined Area fixation duration)	Higher values indicate stronger emphasis on attitude, vertical-state, and lateral/navigation guidance. Lower values may indicate compensatory or reactive monitoring
ASI over-monitoring index	ASI fixation, duration, scanpath, or convex hull normalized by total FA gaze activity	Elevated values may indicate reactive airspeed monitoring, speed fixation, or unstable energy management
AI return-loop rate	Proportion of sequence patterns in which gaze returns to AI after sampling Alt_VSI, TI_HSI, SSI, ASI, RPM, OTW, or Undefined Area	Higher values indicate that attitude remains the central control reference
Coupled guidance–integration rate	Frequency or support of patterns linking AI, Alt_VSI, and TI_HSI	Captures whether attitude, vertical guidance, and lateral/navigation guidance are integrated during the coupled lateral–vertical control phase
ASI fixation-loop index	Frequency or support of patterns such as ASI → AI → ASI, AI → ASI → AI, or ASI → Undefined Area → ASI	Elevated values may indicate reactive speed monitoring or airspeed fixation
Lateral-correction loop index	Frequency or support of patterns such as AI → SSI → TI_HSI, SSI → AI → TI_HSI, and AI → TI_HSI → SSI	Elevated values may indicate reactive lateral or coordination monitoring
Undefined area interruption index	Proportion of sequence patterns containing undefined area	Higher values may indicate scan disruption, weak AOI anchoring, or inefficient visual routing

**Table 5:** Proposed global assessment metrics for the overall approach.

Metric	Definition	Training Interpretation
Overall scan similarity score	Similarity between a pilot’s sequence-support profile and the established reference profile	Provides a global index of how closely a pilot’s scan organization resembles established benchmark
Overall vertical-energy coupling score	Frequency or support of patterns linking Alt_VSI, AI, and ASI, especially Alt_VSI → AI → ASI	Captures whether descent-path monitoring and airspeed management are coupled through attitude control across the full approach
Localized vs. distributed undefined area ratio	Localized undefined area loops, e.g., SSI → Undefined Area → SSI, divided by distributed undefined area patterns involving AI, Alt_VSI, ASI, or TI_HSI	Higher localized ratios may reflect AOI-boundary effects; higher distributed involvement may indicate weak scan anchoring
Full approach fragmentation index	Number of unique recurrent patterns divided by total pattern frequency, or transition entropy across AOIs	Higher values may indicate less stable or less repeatable scan organization across the approach
Phase-transition adaptation score	Change in scan metrics from pre-FAF to FA, such as change in AI-Alt_VSI coupling, ASI monitoring, OTW activity, or undefined area transitions	Captures whether a pilot adapts scan strategy as task demands shift from localizer/altitude management to coupled localizer–glideslope tracking

## Data Availability

The data presented in this study are not openly available due to privacy and ethical restrictions. Requests to access the data should be directed to the lead author.

## Abbreviations

The following abbreviations are used in this manuscript:

AI	Attitude indicator
AOI	Area of interest
Alt_VSI	Altimeter/Vertical speed indicator
ASI	Airspeed indicator
DA	Decision altitude
DH	Decision height
FA	Final approach
FAF	Final approach fix
ILS	Instrument Landing System
KSEA	Seattle–Tacoma International Airport
OTW	Out-the-window
PP	Percentage points
RPM	Tachometer
SD	Standard deviation
SSI	Slip–skid indicator
TI_HSI	Turn indicator/Horizontal situation indicator

## References

[1] Ziv G. (2016). Gaze Behavior and Visual Attention: A Review of Eye Tracking Studies in Aviation. Int. J. Aviat. Psychol..

[2] Peißl S., Wickens C.D., Baruah R. (2018). Eye-Tracking Measures in Aviation: A Selective Literature Review. Int. J. Aerosp. Psychol..

[3] Naeeri S.M., Kang Z., Palma Fraga R. (2022). Investigation of Pilots’ Visual Entropy and Eye Fixations for Simulated Flights Consisted of Multiple Take-Offs and Landings. J. Aviat./Aerosp. Educ. Res..

[4] Lounis C., Peysakhovich V., Causse M. (2021). Visual scanning strategies in the cockpit are modulated by pilots’ expertise: A flight simulator study. PLoS ONE.

[5] Xiong W., Wang Y., Zhou Q., Liu Z., Zhang X., Harris D. (2016). The Research of Eye Movement Behavior of Expert and Novice in Flight Simulation of Landing. Engineering Psychology and Cognitive Ergonomics EPCE 2016.

[6] Kasarskis P., Stehwien J., Hickox J., Aretz A., Wickens C. Comparison of expert and novice scan behaviors during VFR flight. Proceedings of the 11th International Symposium on Aviation Psychology Columbus.

[7] Brams S., Ziv G., Levin O., Spitz J., Wagemans J., Williams A.M., Helsen W.F. (2019). The relationship between gaze behavior, expertise, and performance: A systematic review. Psychol. Bull..

[8] Dehais F., Behrend J., Peysakhovich V., Causse M., Wickens C.D. (2017). Pilot Flying and Pilot Monitoring’s Aircraft State Awareness During Go-Around Execution in Aviation: A Behavioral and Eye Tracking Study. Int. J. Aerosp. Psychol..

[9] Gao S., Wang L. (2024). How flight experience impacts pilots’ decision-making and visual scanning pattern in low-visibility approaches: Preliminary evidence from eye tracking. Ergonomics.

[10] Ayala N., Calderon C.M., Sharma N., Irving E., Cao S., Kearns S., Niechwiej-Szwedo E. Examining the Utility of Blink Rate as a Proxy for Cognitive Load in Flight Simulation. Proceedings of the 2024 Symposium on Eye Tracking Research and Applications (ETRA ’24).

[11] Babu M.D., JeevithaShree D.V., Prabhakar G., Saluja K.P.S., Pashilkar A., Biswas P. (2019). Estimating Pilots’ Cognitive Load from Ocular Parameters Through Simulation and In-Flight Studies. J. Eye Mov. Res..

[12] Fu B., Soriano A.R., Chu K., Gatsby P., Nicolas G. Modelling Visual Attention for Future Intelligent Flight Deck—A Case Study of Pilot Eye Tracking in Simulated Flight Takeoff. Proceedings of the 32nd ACM Conference on User Modeling, Adaptation and Personalization.

[13] Hebbar P.A., Bhattacharya K., Prabhakar G., Pashilkar A.A., Biswas P. (2021). Correlation Between Physiological and Performance-Based Metrics to Estimate Pilots’ Cognitive Workload. Front. Psychol..

[14] Schwerd S., Schulte A. (2024). Triggering Cockpit Alerts Using an Eye-Tracking-Based Measure of Monitoring Performance. Aviat. Psychol. Appl. Hum. Factors.

[15] Fu B., De Jong C., Guardado N.G., Soriano A.R., Reyes A. Event-Driven Predictive Gaze Analytics to Enhance Aviation Safety. Proceedings of the ACM on Human-Computer Interaction.

[16] Fu B., Gatsby P., Soriano A.R., Chu K., Guardado N.G. Towards Intelligent Flight Deck—A Case Study of Applied Eye Tracking in The Predictions of Pilot Success and Failure During Simulated Flight Takeoff. Proceedings of the International Workshop on Designing and Building Hybrid Human–AI Systems (SYNERGY 2024), Co-Located with the 17th International Conference on Advanced Visual Interfaces (AVI 2024), CEUR Vol-3701.

[17] Peysakhovich V., Ledegang W.D., Houben M.M.J., Groen E.L. Classification of flight phases based on pilots’ visual scanning strategies. Proceedings of the 2022 Symposium on Eye Tracking Research and Applications (ETRA ’22).

[18] Zolfaghari F., Papanikou M., Kilikevicius A., Kale U. Deep Learning-Based Classification of Expert and Novice Pilot Performance During Simulated Landings. Proceedings of the 4th International Conference on Cognitive Mobility CogMob 2025.

[19] Rudi D., Kiefer P., Raubal M. (2020). The instructor assistant system (iASSYST)—Utilizing eye tracking for commercial aviation training purposes. Ergonomics.

[20] Arjun S., Hebbar A., Vinod S., Biswas P. Cognitive Load Dashboard for Flight Simulator. Proceedings of the 2022 Symposium on Eye Tracking Research and Applications (ETRA ’22).

[21] Fabre E., Braca P., Peysakhovich V., Dehais F. (2022). A New Interface to Cope with Unreliable Airspeed Indications: A Behavioral and Eye-Tracking Study. Aviat. Psychol. Appl. Hum. Factors.

[22] Fu B. (2026). Eyes on the Approach: Eye Tracking Analysis of Pilot Scan Discipline During Simulated Instrument Landing System Approaches and Implications for Safety. Proceedings of the ACM on Human-Computer Interaction.

[23] Federal Aviation Administration (2012). Instrument Flying Handbook.

[24] Federal Aviation Administration (2018). Instrument Rating-Airplane Airman Certification Standards.

[25] Federal Aviation Administration (2022). Airplane Flying Handbook.

[26] Federal Aviation Administration (2017). Instrument Procedures Handbook.

[27] Fu B., Chu K., Soriano A.R., Gatsby P., Guardado N.G., Jones A., Halderman M. (2025). BEACH-Gaze: Supporting Descriptive and Predictive Gaze Analytics in the Era of Artificial Intelligence and Advanced Data Science. J. Eye Mov. Res..

[28] Bahill T., McDonald J.D. (1983). Frequency limitations and optimal step size for the two-point central difference derivative algorithm with applications to human eye movement data. IEEE Trans. Biomed. Eng..

[29] Krejtz K., Duchowski A., Szmidt T., Krejtz I., González Perilli F., Pires A., Vilaro A., Villalobos N. (2015). Gaze transition entropy. ACM Trans. Appl. Percept..

[30] Doellken M., Zapata J., Thomas N., Matthiesen S. (2021). Implementing innovative gaze analytic methods in design for manufacturing: A study on eye movements in exploiting design guidelines. Procedia CIRP.

[31] Steichen B., Wu M.M.A., Toker D., Conati C., Carenini G. 2014 Te Te Hi, Hi Eye Gaze Sequence Analysis for Informing User-Adaptive Information Visualizations. Proceedings of the 22nd International Conference on User Modeling, Adaptation, and Personalization UMAP 2014.

[32] West J.M., Haake A.R., Rozanski E.P., Karn K.S. eyePatterns: Software for identifying patterns and similarities across fixation sequences. Proceedings of the 2006 Symposium on Eye Tracking Research & Applications.

